# Evaluation of ultrastructural alterations of glomerular basement membrane and podocytes in glomeruli by low-vacuum scanning electron microscopy

**DOI:** 10.1007/s10157-021-02147-z

**Published:** 2021-10-19

**Authors:** Ping Lan, Dedong Kang, Akiko Mii, Yoko Endo, Masako Tagawa, Xiaoyang Yu, Jia Lyu, Liyi Xie, Akira Shimizu, Mika Terasaki

**Affiliations:** 1 Department of Analytic Human Pathology, Nippon Medical School, 1-1-5, Sendagi, Bunkyo-ku, Tokyo, 113-8602 Japan; 2 Department of Nephrology, Kidney Hospital, The First Affiliated Hospital of Xi’an Jiaotong University, Xi’an, China; 3 Department of Nephrology, Nippon Medical School, Tokyo, Japan

**Keywords:** Heymann nephritis, LV-SEM, Membranous nephropathy, Pathology, Renal biopsy

## Abstract

**Background:**

Low-vacuum scanning electron microscopy (LV-SEM) is applied to diagnostic renal pathology.

**Methods:**

To demonstrate the usefulness of LV-SEM and to clarify the optimal conditions of pathology samples, we investigated the alterations of glomerular basement membrane (GBM) and podocytes in control and experimental active Heymann nephritis (AHN) rats by LV-SEM.

**Results:**

On week 15 following induction of AHN, spike formation on GBM with diffuse deposition of IgG and C3 developed. Using LV-SEM, diffuse crater-like protrusions were clearly noted three-dimensionally (3D) on surface of GBM in the same specimens of light microscopy (LM) and immunofluorescence (IF) studies only after removal coverslips or further adding periodic acid-silver methenamine (PAM) staining. These 3D ultrastructural findings of GBM surface could be detected in PAM-stained specimens by LV-SEM, although true GBM surface findings could not be obtained in acellular glomeruli, because some subepithelial deposits remained on surface of GBM. Adequate thickness was 1.5–5 μm for 10% formalin-fixed paraffin-embedded (FFPE) and 5–10 μm for the unfixed frozen sections. The foot processes and their effacement of podocytes could be observed by LV-SEM using 10%FFPE specimens with platinum blue (Pt-blue) staining or double staining of PAM and Pt-blue. These findings were obtained more large areas in 2.5% glutaraldehyde-fixed paraffin-embedded (2.5%GFPE) specimens.

**Conclusion:**

Our findings suggest that LV-SEM is a useful assessment tool for evaluating the alterations of GBM and podocytes in renal pathology using routine LM and IF specimens, as well as 2.5%GFPE specimens.

## Introduction

Low-vacuum scanning electron microscopy (LV-SEM) has a compact body with the advantages of high surface sensitivity and visibility, and recent studies have reported that LV-SEM is useful for ultrastructural assessments of renal pathology using light microscopy (LM) specimens [1, 2]. LV-SEM can evaluate the three-dimensional (3D) ultrastructural changes in the glomerular basement membrane (GBM) and podocytes in formalin-fixed paraffin-embedded (FFPE) LM sections with periodic acid-silver methenamine (PAM) or platinum blue (Pt-blue) staining [3–7]. Using LV-SEM, the characteristic findings of diseases were demonstrated in IgA nephropathy, Alport syndrome, thin basement membrane disease, minimal change nephrotic syndrome, focal segmental glomerulosclerosis, and membranous nephropathy (MN) [3–7]. MN is a common glomerular disease associated with nephrotic syndrome due to the deposit of immune complexes on the GBM [8, 9]. The experimental, active Heymann nephritis (AHN) model has been used as a rat model for human MN [10–14]. In the pathological features of MN, glomerular lesions are characterized by the spike and stippling formation of GBM in PAM staining sections in LM, and the granular deposition of IgG and complement C3 along the capillary walls in immunofluorescence (IF) studies. In transmission electron microscopy (TEM) findings, the subepithelial electron-dense deposits with spike formation and the effacement of foot processes of podocytes were noted. The characteristic alteration of GBM and podocytes develops in the glomeruli in MN.

In this study, to examine whether LV-SEM is able to capture the ultrastructural alterations of GBM and podocytes in MN, and to clarify the optimal conditions of tissue samples for LV-SEM analysis, we examined the morphological alterations of GBM and podocytes in control and experimental AHN rats by LV-SEM using tissue specimens with several different fixation and staining of PAM, Pt-blue, or double staining of PAM and Pt-blue. In addition, we compared directly the GBM findings of LM or IF studies and 3D ultrastructural findings on the GBM surface by LV-SEM, using same specimens of LM and IF studies. In AHN rats, we examined the pathology of the kidneys on week 15 after AHN induction, when the alterations of GBM and podocytes fully developed with massive proteinuria (646.0 ± 168.7 mg/dL). Additionally, we compared the findings of the GBM surface by LV-SEM for PAM staining specimens and by scanning electron microscopy (SEM) or LV-SEM for glomeruli following digestion of all cellular components (acellular glomeruli) that is common technique to ultrastructural evaluation of GBM surface.

## Materials and methods

### Animals

Male Sprague–Dawley (SD) rats (250–300 g) and female Lewis rats (90–140 g) were used for this study. All experiments were carried out by our protocol, which was approved by the animal ethics review committee of Nippon Medical School. Animals were obtained from Sankyo Labo Service Corporation Inc., Japan.

### Induction of active Heymann nephritis

AHN models were induced in female Lewis rats as previously described by Mendrick et al. and Hayashi et al. [13, 14]. Tubular brush border antigen, specifically Fx1A, was prepared from SD rat kidneys according to methods described by Kamata et al. and Edgington et al. [11, 15]. Each rat was immunized subcutaneously with antigen emulsion, which consisted of Fx1A emulsified in complete Freud’s adjuvant (CFA; Sigma, Louis, MO, USA) with 0.5 mg of Mycobacterium tuberculosis H37 Ra (BD, Becton Dickinson Company, Sparks, MD, USA). A 0.1 ml antigen emulsion was injected into each footpad, and an additional 0.05 ml of inactive Bordetella pertussis vaccine containing 4 × 10^9^ organisms (Nacalai Tesque, Kyoto, Japan) was injected subcutaneously into the dorsum of each paw. One booster containing a half dose of the antigen emulsion injection was administered at four weeks after the first immunization. Control rats were only immunized with emulsion saline and CFA.

### Pathological examination

Fifteen weeks after the first immunization in AHN, rats were killed, and renal tissues were divided into several blocks for analysis by LM, IF, TEM, SEM, and LV-SEM.

For the LM study, the tissues were fixed in 10% buffered formalin, dehydrated and embedded in paraffin. These 10%FFPE sections were stained with PAM, hematoxylin–eosin (HE), periodic acid–Schiff (PAS), and Masson’s trichrome (Masson).

For the IF study, frozen specimens were prepared and double immunostained with FITC-conjugated rabbit antibody to rat IgG (1:50; SBA, Birmingham, AL), as well as antibody against rat α3 chain of type IV collagen (major GBM collagen) obtained from Shigei Medical Research Institute (Okayama, Japan) and Texas Red-conjugated rat IgG2b (1:50; Abcam, Cambridge, United Kingdom). The main subclass of deposited IgG on GBM in AHN is known as IgG2a [16], and we recognized that the Texas Red-conjugated rat IgG2b cannot or can only minimally cross react for deposited IgG on GBM in AHN in negative control such as the exclusion of the primary antibody in immunostaining procedure. The sections were observed by Olympus fluorescence microscopy (Olympus, Tokyo, Japan).

For the TEM study, tissues were fixed in 2.5% glutaraldehyde in 0.1 M PBS (pH 7.4), postfixed in 1% osmium tetroxide, and embedded in Epon 812. These 2.5%GFEE ultrathin sections were stained with uranyl acetate and lead citrate or PAM and observed by TEM (H7500, Hitachi, Tokyo, Japan). In addition, we prepared 2.5%GFPE specimens. For TEM observation, 5-μm-thick 2.5%GFPE specimens were deparaffinized, postfixed in 1% osmium tetroxide, and embedded in Epon 812. The ultrathin sections from 2.5%GFPE specimens were also stained with uranyl acetate and lead citrate and observed by TEM.

### Preparation of acellular glomeruli for the evaluation of GBM surface

To evaluate the 3D ultrastructural findings of GBM surface, acellular glomeruli after acellular treatment, that is, digestion of all cellular components, were prepared as described previously [17, 18]. The small renal cortex tissues (2–3 mm^3^) were sequentially exposed to 5 mM EDTA (Sigma Aldrich Corp, St. Louis, MO, USA) for 24 h (h), 3% Triton X-100 (Sigma Aldrich Corp, St. Louis, MO, USA) for 18 h, 0.025% deoxyribonuclease (Worthington Biochemical Crop. Lakewood, USA) in 1 M NaCl for 6 h, and 4% sodium deoxycholate (FUJIFILM Wako Pure Chemical Corp Tokyo, Japan) for 18 h. After t-butyl alcohol vacuum drying, freeze-dried specimens were coated with gold powder and examined by SEM (SU3500, Hitachi, Tokyo, Japan) and LV-SEM (Hitachi Tabletop Microscope TM3030 plus, Hitachi High-Technologies Corp., Tokyo, Japan). Acellular glomeruli stained with uranyl acetate and lead citrate or PAM were also examined by TEM.

### LV-SEM sample preparation

The samples for LV-SEM were prepared as described previously [1–7]. Both 10%FFPE sections and unfixed frozen sections stained with PAM and/or Pt-blue (TI blue; Nisshin EM Co. Ltd, Tokyo, Japan) were observed by LV-SEM. The relationship between the LM findings and the ultrastructural 3D findings of GBM in the same portion of glomerulus was observed by LM and LV-SEM. After observation of the 10%FFPE section with PAM staining by LM, 3D ultrastructural GBM surface findings were assessed for same specimen by LV-SEM after only removing the coverslip. After observation of PAM-stained specimens by LV-SEM, these dried specimens were remounted with coverslips, and the quality of PAM staining before and after LV-SEM observation was compared. The relationship between IF findings and the ultrastructural 3D findings of GBM in the same portion of glomerulus was observed using same frozen IF specimens. After observation of IF findings for the frozen sections with double immunostaining for IgG and α3 chain of type IV collagen, the coverslip was removed, and the frozen sections were fixed with 10% buffered formalin, stained with PAM, and observed by LV-SEM. To determine the adequate thickness of specimens with PAM staining for LV-SEM, different thicknesses (2 μm, 5 μm, 10 μm, and 20 μm) were prepared for frozen sections with PAM staining as well as for 10%FFPE sections with PAM staining. We also evaluated the 3D ultrastructural alterations of GBM surface and podocytes in control and AHN rats using 10%FFPE LM specimens, 2.5%GFEE TEM semithin sections, and 2.5%GFPE specimens with PAM staining, Pt-blue staining or double staining of PAM and Pt-blue.

## Results

### Findings of GBM in 10%FFPE sections with PAM staining by LM and LV-SEM

**Fig. 1:**
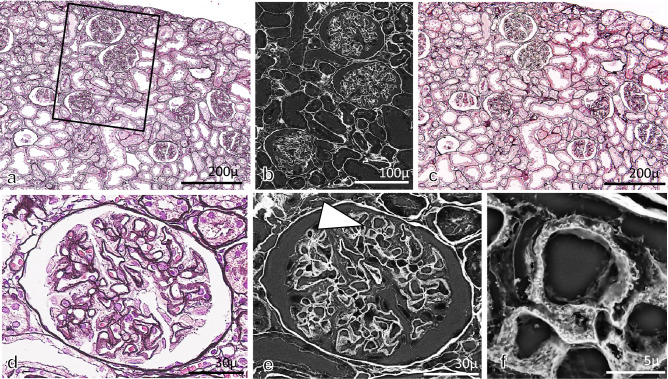
Pathological findings of LM and LV-SEM in AHN rats. The same areas of kidney were examined by LM and LV-SEM. After observation by LM (**a**), PAM-stained 10% FFPE specimens after denuded coverslips can be evaluated 3D ultrastructurally by LV-SEM (**b**). After observation by LV-SEM, the denuded dry specimens were remounted with coverslips (**c**). The quality of PAM staining by LM observation was the same as that before removal of the coverslips. The pathological findings of the same glomeruli could be evaluated by LM (**d**) and LV-SEM (**e**). In addition, the area of the glomerulus (arrow in **e**) could be observed with high magnification by LV-SEM (**f**)

**Fig. 2:**
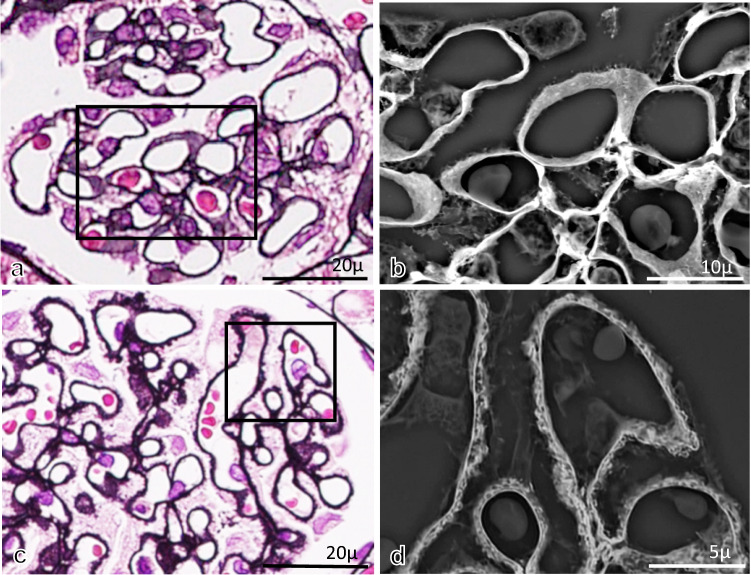
Pathological findings of LM and LV-SEM. In the control rats (**a**, **b**), smooth GBM was noted in LM (**a**), and a smooth GBM surface was also evident in LV-SEM findings (**b**). In the glomerulus in AHN rats, small and irregular spike formations were seen in LM (**c**), and spikes, stippling, and crater-like holes on the GBM surface were evident in LV-SEM findings (**d**)

Morphological alterations of glomeruli in AHN rats were evaluated in the 10%FFPE LM specimens with PAM staining by LM (Fig. 1a). Then, following removal of the coverslips, the denuded dry sections were observed by LV-SEM (Fig. 1b). After observation by LV-SEM for denuded dry specimens with PAM staining, specimens were remounted with coverslips. The quality of PAM staining remained the same as before removal of the coverslips, even after LV-SEM observation (Fig. 1c). This method enables us to identify the relationship between LM findings and 3D ultrastructural findings by LV-SEM in exactly the same area in the same specimen (Fig. 1d–f). Indeed, in control rats (Fig. 2a, b), the surface of the GBM was smooth, as shown by LM and LV-SEM. In the specimens from rats in the AHN model, small and irregular spike formations were noted along the glomerular capillaries by LM (Fig. 2c), and the spike and stippling formations on GBM were clearly noted by LV-SEM (Fig. 2d).

### The relationship between the findings of GBM by IF and LV-SEM

**Fig. 3:**
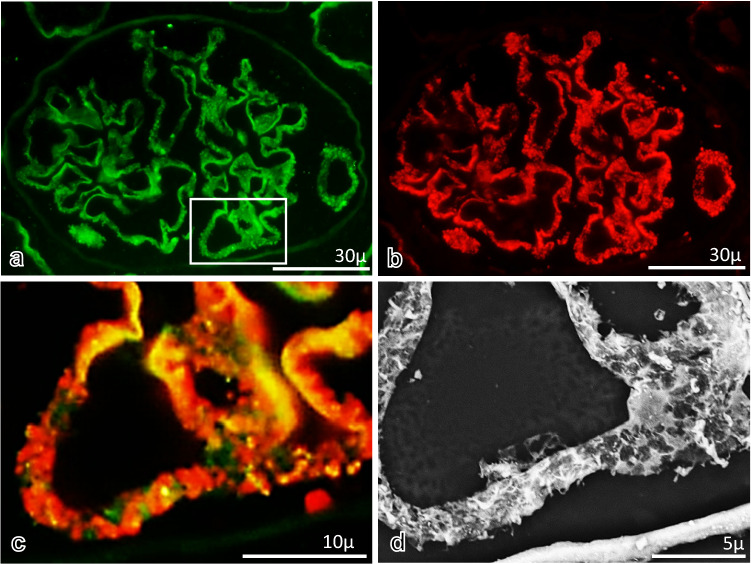
The comparison of the findings of IF and LV-SEM. In the IF study of double immunostaining against IgG (**a**) and the α3 chain of type IV collagen (**b**), the deposition of IgG was seen as a diffuse granular pattern on GBM with the irregular expression of the α3 chain of type IV collagen, indicating irregular morphological alterations of GBM with IgG deposition (**c**: merged image **a** and **b**). After observation by IF, the frozen sections after removal of coverslip were fixed with 10% buffered formalin, stained with PAM, and evaluated by LV-SEM. In this method, the same portion of glomerulus could be observed by IF and LV-SEM. In the high magnification LV-SEM image, the GBM surface could be evaluated 3D ultrastructurally by LV-SEM, and multiple, irregular crater-like holes were clearly confirmed on the surface of the GBM (**d**)

In AHN rats, we performed double immunostaining with anti-rat IgG antibodies (IgG deposition) and anti-α3 chain of type IV collagen using frozen sections. In the IF study, diffuse granular deposition of IgG was noted along the glomerular capillaries (Fig. 3a). In addition, irregular expression of the α3 chain of type IV collagen, which is one of the main components of GBM, was noted (Fig. 3b). The merged images of the deposition of IgG and the expression of the α3 chain of type IV collagen indicated that the irregular expression of the α3 chain of type IV collagen was associated with the deposition of IgG (Fig. 3c). In LV-SEM findings with higher magnification of the same area, prominent crater-like and stippling lesions were found on the GBM (Fig. 3d). After the evaluation of IF, it is possible that ultrastructural alterations of the GBM surface could be observed in the same area by LV-SEM, following removal of coverslip and PAM staining.

### Adequate thickness of specimens with PAM staining for LV-SEM observation

**Fig. 4:**
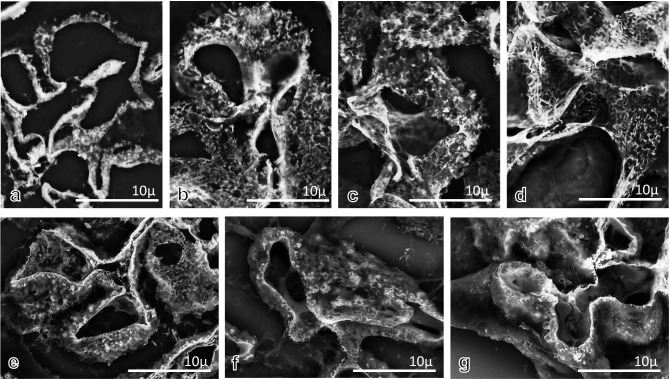
Thickness of specimens adjusted for evaluation of the GBM surface by LV-SEM. LV-SEM findings were compared in frozen sections of different thicknesses (**a**–**d**; PAM stain, **a**: 2 μm, **b**: 5 μm, **c**: 10 μm, **d**: 20 μm) and 10% FFPE LM sections (**e**–**g**: PAM stain, **e**: 1.5 μm, **f**: 5 μm, **g**: 10 μm). In the frozen specimens, when the thickness of the specimen was 2.0 μm (**a**), it was difficult to obtain 3D images of the GBM surface in large areas. For frozen specimens, a thickness between 5 μm (**b**) and 10 μm (**c**) was optimal. LV-SEM provided 3D images of the GBM surface in PAM-stained LM sections when the specimen thickness was 1.5 μm (**e**) and 5.0 μm (**f**). At a thickness of 10 μm (**g**), the quality of PMA staining deteriorated, and detailed information could not be detected. For LM specimens, a thickness between 1.5 and 5.0 μm was optimal

To examine the adequate thickness of specimens for evaluation of the GBM surface by LV-SEM, frozen sections and 10%FFPE LM sections with different thicknesses and PAM staining were observed by LV-SEM (Fig. 4a–g). At all levels of thickness in frozen and 10%FFPE sections, 3D ultrastructural findings could be obtained. However, it was difficult to obtain 3D images of the surface of GBM in large areas in the 2.0 μm frozen sections (Fig. 4a). In the PAM-stained sections for the examination of the GBM surface, the adequate thickness of specimens was 5–10 μm in frozen sections (Fig. 4b, c) and 1.5–5 μm in 10%FFPE sections (Fig. 4e, f).

### Ultrastructural alterations of GBM in acellular glomeruli by SEM, LV-SEM, and TEM

**Fig. 5:**
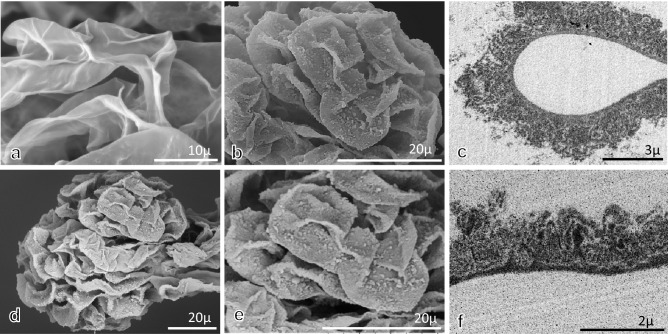
Ultrastructural alterations of the GBM surface in acellular glomeruli in control and AHN rats. To examine the ultrastructural alterations of the GBM surface, acellular glomeruli from control (**a**) and AHN rats at week 15 (**b**–**f**) were examined by SEM (**a**, **b**), LV-SEM (**d**, **e**), and TEM (**c**: uranyl acetate and lead citrate staining, **f**: PAM staining). In control glomeruli, the GBM surface was smooth (**a**). In the glomeruli from AHN rats, an irregular, rough surface structure was noted on the surface of GBM by SEM (**b**). The similar quality of ultrastructural 3D findings was obtained by LV-SEM (**d**, **e**). Spike and crater-like holes could not be detected clearly in acellular glomeruli. For the TEM findings in the specimens with uranyl acetate and lead citrate staining (**c**) and PAM staining (**f**), podocytes and endothelial cells were denuded, but diffuse subepithelial deposits were remained on GBM

To observe the findings of the GBM surface, acellular glomeruli were prepared following digestion of all cells, including podocytes, endothelial cells, and mesangial cells (acellular treatment for glomeruli) and observed by SEM, LV-SEM, and TEM. Evaluation by SEM showed a uniformly smooth GBM surface in acellular glomeruli in control rats (Fig. 5a). Evaluation by TEM indicated that the podocytes and endothelial cells disappeared, and only the GBM remained in the glomerular capillaries. In acellular glomeruli in AHN rats examined by SEM, the surface of the GBM showed diffusely rough surfaces and varying granular structures without podocytes (Fig. 5b). The irregular, rough surface with granular structure of GBM was also identified by LV-SEM (Fig. 5d, e) and determined to be similar in quality until × 18,000 magnification when compared to high-resolution SEM. In acellular glomeruli in AHN rats examined by TEM, some subepithelial deposits remained on the GBM (Fig. 5c, f). This indicated that the GBM surface after acellular digestion for glomeruli in this study could not demonstrate the true surface of GBM due to modification by subepithelial deposits on GBM.

### Evaluation of findings of podocytes in control and AHN rats

**Fig. 6:**
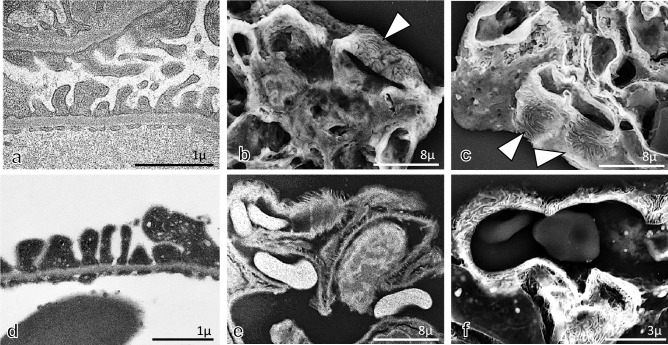
The findings of podocytes in glomeruli from control rats. In the TEM findings (**a**), podocytes with foot processes, GBM, and endothelial cells with fenestration were seen in glomerular capillary walls. In the LV-SEM findings in the 10%FFPE LM specimens with Pt-blue staining (**b**) or double staining of PAM and Pt-blue (**c**), the foot process of podocytes covering the GBM could be confirmed in localized areas (arrowhead). In ultrathin sections from 2.5%GFPE specimens with uranyl acetate and lead citrate and observed by TEM (**d**), foot processes of podocytes, GBM, and fenestra of endothelial cells could be detected in glomerular capillary walls. In the LV-SEM findings in the 2.5%GFPE specimens with Pt-blue staining (**e**) or double staining of PAM and Pt-blue (**f**), the foot process of podocytes covering the GBM could be confirmed as small protrusions on GBM or the foot process interdigitation structure on GBM

**Fig. 7:**
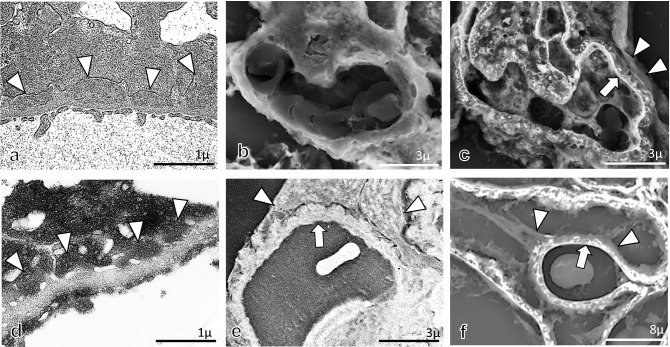
The findings of podocytes in glomeruli from AHN rats. In the TEM findings (**a**), diffuse subepithelial deposits (arrowheads) on the GBM were seen with the effacement of foot processes of podocytes and fenestrated glomerular endothelial cells. In the LV-SEM findings in the 10%FFPE LM specimens with Pt-blue staining (**b**), although GBM and podocytes could not be clearly differentiated, thickened glomerular capillary wall was demonstrated. In the LV-SEM findings in the 10%FFPE LM specimens with double staining of PAM and Pt-blue (**c**), irregular spike formation of GBM (arrow) and podocytes with irregular effacement of foot processes (arrowhead) were noted. In the TEM findings of ultrathin sections from 2.5%GFPE specimens with uranyl acetate and lead citrate and observed by TEM (**d**), diffuse subepithelial deposits (arrowheads) on the GBM were seen with the effacement of foot processes of podocytes and fenestrated glomerular endothelial cells. For the LV-SEM findings in the 2.5%GFPE specimens with Pt-blue staining (**e**), although subepithelial deposits were not clearly shown, thickening of the GBM (arrow) and effacement of the foot processes of podocytes (arrowhead) were demonstrated. In the LV-SEM findings in the specimen with double staining of PAM and Pt-blue (**f**), both GBM with spike formation (arrow) and the effacement of foot processes of podocytes (arrowhead) were clearly observed

In TEM observation, the foot processes of podocytes on GBM in control rats and subepithelial electron-dense deposits on GBM with small spike formations and effacement of the foot process of podocytes were noted in 2.5%GFEE and 2.5%GFPE ultrathin sections (Figs. 6a, d, 7a, d), although these findings were more clearly observed in 2.5%GFEE sections. In the LV-SEM findings of 10%FFPE and 2.5%GFPE specimens with Pt-blue staining or double staining of PAM and Pt-blue, foot processes of podocytes showing small protrusions and interdigitation could be recognized along the GBM in control rats (Fig. 6b, c, e, f), although these findings could be obtained in more large areas in 2.5%GFPE specimens. In 10%FFPE specimens with Pt-blue staining in AHN rats (Fig. 7b), thickening glomerular capillary walls were detected without the differentiation clearly between subepithelial deposits, GBM, and podocytes. In 2.5%GFPE specimens with Pt-blue staining in AHN rats (Fig. 7e), LV-SEM findings showed the effacement of foot processes of podocytes on obviously thickened GBM, although they did not differentiate between subepithelial deposits and GBM components. In 10%FFPE and 2.5%GFPE specimens with double staining of PAM and Pt-blue in AHN rats (Fig. 7c, f), LV-SEM showed uneven crater-like and stippling lesions on GBM, and the effacement of foot processes of podocytes was detected, although these findings were noted as more clearly and in large areas in 2.5%GFPE sections when compared with 10%FFPE specimens. Epon 812-emdedded 2.5-μm-thick TEM semithin sections could not be stained successfully by PAM, Pt-blue, or both PAM and Pt-blue, and the specimens of 2.5%GFEE TEM semithin sections, therefore, could not be used for examination by LV-SEM (data not shown).

## Discussion

In this study, we demonstrated that LV-SEM could be used for the detection of ultrastructural alterations of GBM and podocytes in the pathological diagnosis of renal biopsies. In renal biopsy samples, the 10%FFPE LM sections, unfixed frozen IF sections, and 2.5%GFPE sections could be used for LV-SEM analysis after staining of PAM, Pt-blue, and double staining of PAM and Pt-blue. In addition, LV-SEM analysis could assess the relationship between the LM or IF findings and 3D ultrastructural findings by LV-SEM analysis using the same LM or IF specimens. The alterations of podocytes could be evaluated by LV-SEM using 10%FFPE sections and 2.5%GFPE sections, although large areas with high quality of the alteration of podocytes could be obtained using 2.5%GFPE sections. We believe that LV-SEM can be widely used as an efficient assessment tool for the ultrastructural findings of renal pathology.

The pathological findings of each LM, IF, and TEM in renal biopsy provide essential and important information for the accurate pathological diagnosis of renal diseases. However, we have sometimes experienced that LM, IF, and/or TEM studies cannot be performed due to the lack of glomeruli in renal tissue samples, especially in small TEM samples. Using LV-SEM, ultrastructural findings can be obtained from LM and IF specimens in renal biopsy. In PAM-stained sections for LM, the 3D structural surface of the GBM could be observed by LV-SEM only after removal of the cover glasses [3–7]. Indeed, in AHN rats, uneven crater-like and stippling lesions were clearly found on the GBM surface by LV-SEM. It is of particular significance and utility that the LM findings and ultrastructural findings with high magnification can be compared using LV-SEM when atypical and/or uncertain findings are observed by LM. After observation by LV-SEM using denuded PAM-stained LM specimens, we confirmed that the quality of PAM staining in specimens with remounting coverslips was same as before removal of the coverslips.

In this study, we obtained foot process interdigitation in 10%FFPE sections, even in limited areas of glomerular capillaries. Recent articles by Inaga et al. and Okada et al. [3, 5] demonstrated that foot process interdigitation of podocytes in glomeruli is well preserved in 10%PPFE sections in clinical renal biopsies. We considered the size of tissue samples to be important for the first fixation in 10% buffered formalin because the tissue size from the experimental rats in this study is a relatively large to examine large area of the kidney. Using 2.5%GFPE specimens with Pt-blue staining, the foot processes of podocytes in control rats and the effacement of the foot processes of podocytes in AHN rats could be observed. In 2.5%GFPE sections with double staining of PAM and Pt-blue, ultrastructural alterations of the GBM surface and podocytes could be observed by LV-SEM. LV-SEM was capable of stably evaluating large areas of ultrastructural alterations of GBM and podocytes using 2.5%GFPE specimens with double staining of PAM and Pt-blue.

The 2.5%GFEE TEM semithin sections could not be stained successfully with PAM, Pt-blue, and double staining of PAM and Pt-blue, and thus could not be used for LV-SEM, although high quality TEM findings could be obtained using 2.5%GFEE sections, and PAM staining could be performed for ultrathin TEM sections. On the other hand, 2.5%GFPE specimens could not be used for the usual LM study because the quality of staining of HE, PAS, Masson, and PAM was not adequate for their evaluation (data not shown). However, using 2.5%GFPE specimens, the foot processes of podocytes, electron-dense deposits, and fenestrae of endothelial cells in glomerular capillaries could be evaluated in the TEM study, and the alteration of podocytes and GBM could be assessed in the LV-SEM study. Depending on the purpose of the study, especially in experimental animal studies, it may be necessary to select the process for tissue making, such as embedding in Epon 812 or paraffin. In this study, we could not decide on the best fixation and best sample size in LV-SEM analysis, and these might be dependent on the aim of the examination of pathological findings by LV-SEM. The findings of podocytes could be observed by LV-SEM in 10%FFPE samples, even in limited areas, may indicate that the good condition of frozen samples and good fixation of 10%FFPE samples are important to obtain good ultrastructural findings of GBM and podocytes in LV-SEM observation.

For the analysis of the surface of GBM, acellular technique for glomeruli has been performed [17, 18]. Bonsib et al. [17] demonstrated that acellular technique for glomeruli is useful for the analysis of 3D ultrastructural GBM surface of several glomerular diseases, including MN. Hironaka et al. [18] demonstrated that some subepithelial deposits remained on GBM with thin fibril spikes in AHN rats after acellular technique. In this study, the true surface findings of GBM could not be evaluated in AHN rats due to subepithelial deposits remaining on the GBM after acellular treatment. Furthermore, this study demonstrated that PAM-stained specimens observed by LV-SEM is easy and simple method to analysis for 3D ultrastructural findings of GBM surface.

LV-SEM is capable of using the same frozen sections following an IF study [7]. In frozen sections, the immune reactivity for antigens is commonly well preserved, and an IF study is performed to detect the presence and localization of specific proteins in tissues. This suggests that combining IF studies and LV-SEM can considerably aid in localizing specific proteins in ultrastructural findings. Usually, the localization of specific proteins in ultrastructural findings is commonly performed by immunoelectron microscopy (Immuno-EM). Immuno-EM is able to detect specific proteins with high resolution by EM. However, it is difficult to apply immuno-EM for the routine pathological diagnosis of renal biopsy due to poor antigen preservation in 2.5%GFEE specimens. It is possible that the utilization of high-resolution imaging by LV-SEM, combined with specific protein detection by IF, may be useful in evaluating the relationship between the specific protein and ultrastructural findings.

## Conclusion

In this study, we determined that LV-SEM can be used to evaluate the ultrastructural alterations of GBM and podocytes in the pathological diagnosis of renal biopsy. The 10%FFPE LM section and frozen IF section from renal biopsy and 2.5%GFPE specimens can be used for LV-SEM observation with PAM and/or Pt-blue staining. To detect clear ultrastructural findings as in TEM, further studies are necessary to determine more better conditions in LV-SEM analysis.
